# Comparison of Gut Bacterial Communities of Fall Armyworm (*Spodoptera frugiperda*) Reared on Different Host Plants

**DOI:** 10.3390/ijms222011266

**Published:** 2021-10-19

**Authors:** Dongbiao Lv, Xueying Liu, Yanlu Dong, Zizheng Yan, Xuan Zhang, Ping Wang, Xiangqun Yuan, Yiping Li

**Affiliations:** 1 Key Laboratory of Integrated Pest Management on Crops in Northwestern Loess Plateau, Ministry of Agriculture, College of Plant Protection, Northwest A&F University, Yangling, Xianyang 712100, China; dongbiao_0613@163.com (D.L.); xueyingliu@nwsuaf.edu.cn (X.L.); yanludong@nwsuaf.edu.cn (Y.D.); 2713649881@nwsuaf.edu.cn (Z.Y.); zhangxuan@nwsuaf.edu.cn (X.Z.); 2 Key Laboratory of Plant Protection Resources and Pest Management, Ministry of Education, College of Plant Protection, Northwest A&F University, Yangling, Xianyang 712100, China; 3 Department of Entomology, Cornell University, Geneva, NY 14456, USA; pingwang@cornell.edu

**Keywords:** *Spodoptera frugiperda*, host species, gut microbiota, 16S rRNA, host adaptation

## Abstract

*Spodoptera frugiperda* is a highly polyphagous and invasive agricultural pest that can harm more than 300 plants and cause huge economic losses to crops. Symbiotic bacteria play an important role in the host biology and ecology of herbivores, and have a wide range of effects on host growth and adaptation. In this study, high-throughput sequencing technology was used to investigate the effects of different hosts (corn, wild oat, oilseed rape, pepper, and artificial diet) on gut microbial community structure and diversity. Corn is one of the most favored plants of *S. frugiperda*. We compared the gut microbiota on corn with and without a seed coating agent. The results showed that Firmicutes and Bacteroidetes dominated the gut microbial community. The microbial abundance on oilseed rape was the highest, the microbial diversity on wild oat was the lowest, and the microbial diversity on corn without a seed coating agent was significantly higher than that with such an agent. PCoA analysis showed that there were significant differences in the gut microbial community among different hosts. PICRUSt analysis showed that most of the functional prediction categories were related to metabolic and cellular processes. The results showed that the gut microbial community of *S. frugiperda* was affected not only by the host species, but also by different host treatments, which played an important role in host adaptation. It is important to deepen our understanding of the symbiotic relationships between invasive organisms and microorganisms. The study of the adaptability of host insects contributes to the development of more effective and environmentally friendly pest management strategies.

## 1. Introduction

The fall armyworm (FAW), *Spodoptera frugiperda* (J.E. Smith) (Lepidoptera: Noctuidae), is a highly polyphagous invasive pest originating from tropical and subtropical regions of America [1,2,3,4,5]. It has a wide suitable region, wide host range, strong reproductive potential, fast diffusion rate, and produces heavy burst damage. In 2016, FAWs invaded various African countries [6,7], and in 2018, they invaded Southeast Asian countries [8]. In 2019, FAW invaded Korea and Japan [9]. By mid-December 2018, the first invasive species of FAW invaded Yunnan Province in western China [10], and by 31 August 2020, they were rapidly spreading across 27 provinces and 1338 counties [11]. FAW were able to reduce corn production by 34% in Brazil, causing losses of USD 4 billion per year [11]. The larvae of FAW can damage 353 species, 227 genera, and 76 families of plants [12]. Gramineous crops such as corn are their preferred hosts; domestic studies have shown that the FAW also feeds on oilseed rape, pepper, rapeseed, wild oat, sorghum, wheat, soybean, potato, and other vegetable crops [8,13]. The multi-host damage of FAW has caused serious economic loss to crops [14]. However, frequent use of pesticides causes crop pesticide residues, increases the risk of environmental pollution, and affects the population dynamics of beneficial insects and natural enemies [14,15,16]. Control strategies governing the FAW have become a most difficult problem in maintaining agricultural production and ecological balance stability [14,15,17]. Therefore, more effective green control methods are urgently needed in order to enact prevention and control mechanisms for FAW [15].

The gut microbial community plays an important role in the biological system of the host, which is defined as a virtual organ and plays an indispensable role in the growth and reproduction of the host [18,19,20]. The symbiotic microbial community participates in insect diversity and evolution to a certain extent, affects insect performance, and improves digestive system function and nutrient availability in the host [17,21,22,23,24]. The insect-related microbial community changes dynamically, and adapts to various stress factors. Under natural conditions, insects and their related microbial communities can adapt under stress; this can shape new adaptive abilities through feeding habits and host factors, and reduce survival stress [18,25,26,27]. It has been reported that some insect microbial communities affect the adaptation of insect-feeding species, causing changes in insect behavior and population ecology [28,29,30]. Such typical functions have been reported in pests such as the FAW, where the insect microbial community plays an important role in the natural evolutionary tendency of the biological ecology [17,31]. The effects of gut microbes on insects are relevant in the fields of medicine, agriculture, and ecology. Insect–microbial interactions have serious impacts on natural ecosystems and agriculture. The study of the gut microbiome associated with insect hosts provides a basis for subsequent functional studies. The gut microbiome of insects is affected by many factors, such as feeding, sex, and achieved developmental stages [17,32,33].

In recent years, more insect microbial communities have been discovered and studied. Studies have been reported in honeybees [34,35,36,37], fruit flies [38,39,40], mosquitoes [41,42], *Dectes texanus* [43], and termites [44,45], as well as in lepidopteran pests, such as *Grapholita molesta* [18,46,47,48], *Carposina sasakii* [46,47], and *Cydia pomonella* [47]. The gut microbial community of the honeybee has stable species and structure, and there are many unique microbial types in different regions of *Apis mellifera*. There are many similarities between the honeybee gut and the human gut in the study of the interactions between the honeybee gut microbial community and host. Thus, the honeybee plays an important role in medicine, agriculture, and ecology, and is an important model organism for the study of gut microbial communities [34,35,36,37]. When *Drosophila* species feed on different hosts, the gut microbial community affects nutrient absorption and hormone release, thus affecting growth and development [38,39,40]. There were significant changes in the microbial communities of different mosquitoes at the same site, showing the important role of gut microbes in mosquito reproduction [41,42]. The gut microbial community of *Blattella germanica* is highly dynamic, because the microbial community restructures for a specific dietary pattern within a short period of time. Flexible changes in the gut microbial community may be due to diverse feeding [49]. Compared with *D. texanus* fed on different hosts, 514 unigenes were differentially expressed in larvae feeding on soybeans [43]. Host specificity in biodiversity highlights the different degrees and interactive diversity across the levels of biodiversity in the system. Host digestion strategies define the structural and functional potential that drive the gut microbial community [44,45]. OTUs (operational taxonomic units) and species abundance were significantly different in different instars across the whole life cycle of *G. molesta*. At the same time, the variable environment affected their gut microbial communities [18,46,47,48].

In order to study the changes in the gut microbial community of the FAW when feeding on different hosts, Illumina sequencing of the 16S rRNA gene was used to elucidate the microbial community structure of the FAW when feeding on an artificial diet, corn, wild oat, oilseed rape, and pepper. To further determine the effects of different treatments on the gut microbial community of the same host, corn was divided into two groups: with and without a seed coating agent. Our aim was to study the effects of host plants on the gut microbial community of the FAW, which is closely related to the study of the biology and ecology of host insects, and to lay a foundation for the study of green and efficient control measures.

## 2. Results

### 2.1. Analysis of rRNA Sequencing Results

High-throughput 16S rRNA sequencing was collected for the gut microbial community composition of 18 midgut samples of FAW fed on 5 different host plants—corn (without a seed coating agent (CA), and with a seed coating agent (CB)), wild oat (WO), oilseed rape (OR), and pepper (PP)—and an artificial diet (AD). Clean tag data from the original data were distributed between 33,892 and 71,996 after quality control. Removing chimeras yielded valid tag data distributed between 30,933 and 67,211. The proportion of valid tags ranged from 84.48 to 95.50%. The average length of valid tags ranged from 374.72 to 423.16 bp. The number of OTUs in each sample ranged from 642 to 2235 (Table S1). Detected gut microorganisms were classified into 30 phyla, 76 classes, 167 orders, 321 families, 755 genera, and 321 species. The alpha diversity index of the sample was analyzed (Table S2). According to the Chao1 index and Shannon index dissolution curves, the quality of species abundance and distribution uniformity of the samples were high, and the sequencing volume and depth of the samples were large enough (Figure S1). Almost all of the Good’s coverage of the samples exceeded 0.9900 (except for the OR of 0.9800), which was equivalent to the number of observed species in each group, indicating that the sequencing integrity was good, and almost all of the species in the samples were detected (Table S2). In general, with regard to the quality control of tags, alpha diversity, and the annotation of OTUs at each classification level, it can be said that the sampling and sequencing operations in each treatment group were proper in this experiment, with reliable data obtained that will be valuable for in-depth follow-up analysis.

### 2.2. Comparison of the Gut Microbial Community Dynamics across Different Hosts

**Figure 1:**
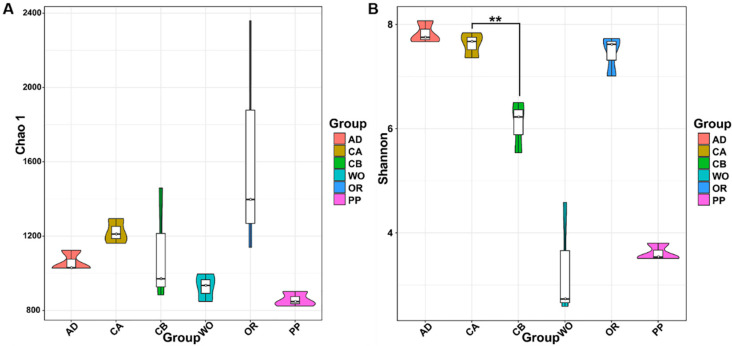
Violin plot of OTU diversity index comparison between groups of (**A**) Chao1 and (**B**) Shannon. The abbreviations in the figure represent different hosts. Asterisks indicate significant differences between hosts (**, *p* < 0.01; independent samples *t*-test).

Alpha diversity analysis reflects the degree of species diversity in the biological environment. The alpha diversity index in the sequencing results reflected the differences in gut microbial communities among and within the samples. They included Chao1, Shannon, Simpson, PD_whole_tree, and Good’s coverage (Table S2). The Chao1 index reflected the abundance of microorganisms, while the Shannon and Simpson indices reflected the diversity of microorganisms. The microbial communities associated with different hosts were diverse. Oilseed rape had the highest Chao1 abundance values, whereas pepper and wild oat held the lowest bacterial abundance and Shannon diversity, respectively (Figure 1). The microbial abundance of corn without a seed coating agent was higher than that of corn with a seed coating agent, and the bacterial diversity of the former was significantly higher than that of the latter (F = 23.213; df = 1.4; *p* = 0.009) (Figure 1B).

**Figure 2:**
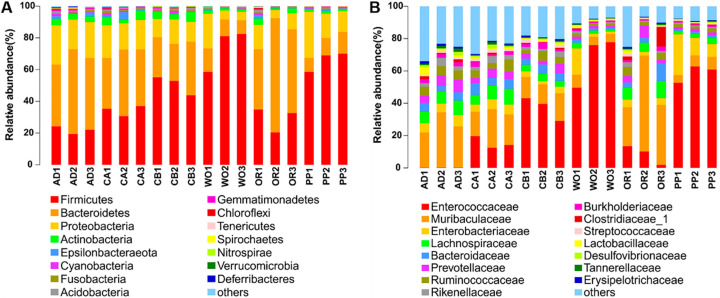
Top 15 in relative abundance in microorganism composition at the (**A**) phylum and (**B**) family levels. Columns represent different samples, different colors represent different annotated information, and “others” represent all species except those annotated above.

We performed identification and in-depth analysis of gut microbial communities at both the phylum and family taxonomic levels. The top 15 in the relative abundance of the phyla and families were analyzed across all samples (Figure 2). At the phylum level, Firmicutes, Bacteroidetes, Proteobacteria, and Actinobacteria were ubiquitous in all samples, and the total relative abundance of the species ranged from 93.47 ± 0.88% to 97.96 ± 0.42% in different hosts (Figure 2A and Table 1). Firmicutes were the largest phylum in relative abundance, especially for wild oat and pepper, accounting for 74.05 ± 7.76% and 65.84 ± 3.65%, respectively, which was significantly higher than that in other host samples, and relatively lower than for artificial diet and oilseed rape (F = 23.695; df = 5.12; *p* < 0.001). Bacteroidetes accounted for 54.48 ± 9.88% and 45.82 ± 4.22% on oilseed rape and artificial diet, respectively, which was significantly higher than that for wild oat and pepper (F = 13.650; df = 5.12; *p* < 0.001). The relative abundance of Proteobacteria and Actinobacteria in each host was 10.30 ± 2.97% to 22.16 ± 1.80% and 1.38 ± 0.13% to 4.55 ± 0.88%, respectively. At the family level, Enterococcaceae and Muribaculaceae are two very special microbial families (Figure 2B). The relative abundance of Enterococcaceae on wild oat and pepper was 67.76 ± 9.12% and 58.71 ± 3.11%, respectively, which was significantly higher than that on other host samples—especially on artificial diet and oilseed rape, where the proportion was 0.27 ± 0.01% and 8.53 ± 3.44%, respectively (F = 36.394; df = 5.12; *p* < 0.001) (Figure 2B and Table 1). The fact that the relative abundances of important families varied by hundreds of times in the same batch of experiments is a particularly interesting point from the study. Muribaculaceae were as high as 40.10 ± 1.08% on oilseed rape, 6.09 ± 0.97% on wild oat, and 6.49 ± 0.85% on pepper (Table 1). There were significant differences between different hosts (F = 7.942; df = 5.12; *p* < 0.05). In addition, Enterobacteriaceae were 14.76 ± 5.33% on pepper and 3.35 ± 0.60% on corn with a seed coating agent, which is also a research point worth thinking about. Enterococcaceae on corn with a seed coating agent were more abundant than on corn without a seed coating agent. However, Muribaculaceae, Enterobacteriaceae, and Lachnospiraceae were more abundant on the latter than on the former. There were differences in the relative abundance of the main microorganisms between the two kinds of corn (F = 10.308; df = 5.12; *p* < 0.05).

**Figure 3:**
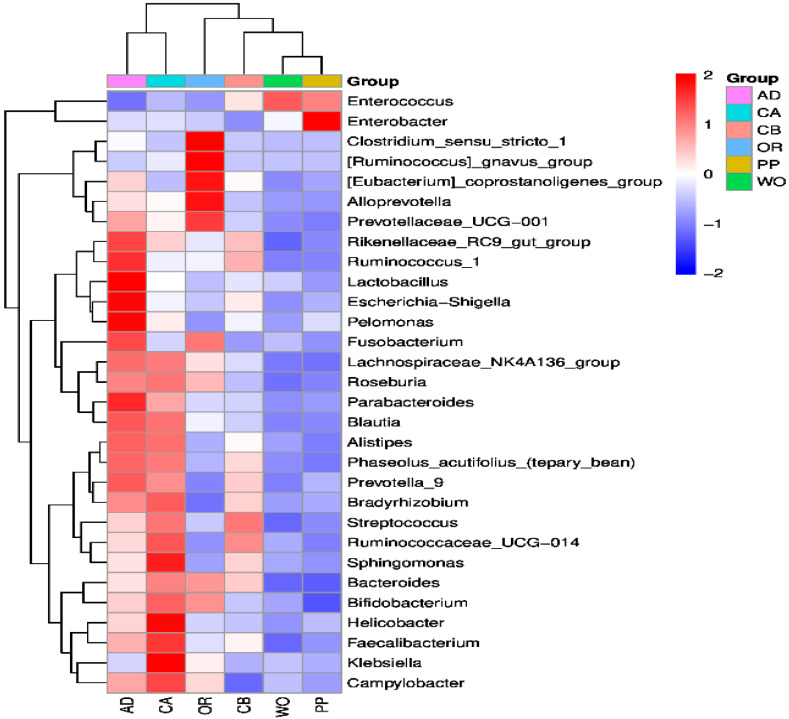
Heatmap of the top 30 genera in terms of relative abundance at the genus level of each host. Columns represent different host samples, while rows represent bacterial ASV at the generic level. A cluster tree of sample microorganisms and cluster analysis of each genus are shown at the top and left, respectively. The color scale represents the normalized values of relative abundances by log10.

In order to more clearly understand the dynamic pattern of gut microbiota feeding on different hosts, combined with the above analysis at the phylum and family levels, the datasets of bacteria with relative abundance in the top 30 at the genus level were selected for the heatmap. The ANOVA method was used to compare the significance of differences between samples. It was found that three samples from each host could cluster in one branch, and similar hosts could also cluster in one branch (Figure S2). The results indicated that the inter-host settings and intra-host repeatability of the samples were suitable. The similarities of microbial communities between the groups were measured via the Bray–Curtis distance calculation method (Figure 3). Columns represent different host samples, while rows represent genera ASV. The microbial communities on wild oat and pepper were similar in composition, while corn with a seed coating agent and oilseed rape were clumped together into one large branch of different branches. The microbial communities on the artificial diet and corn without a seed coating agent were similar in composition and clustered together. The differences between the two branches are obvious. Only *Enterobacter* had a very high abundance on pepper, while other microbes had a very low abundance. Similarly, only *Enterococcus* had a very high abundance on wild oat, while other microbes had a very low abundance on wild oat. *Enterococcus* and *Klebsiella* had high abundance only on pepper and corn without a seed coating agent, respectively. *Clostridium* and *Ruminococcus* were very abundant only on oilseed rape. *Lactobacillus*, *Escherichia-Shigella*, and *Pelomonas* were found to be highly abundant only on artificial diets. *Sphingomonas*, *Helicobacter*, *Faecalibacterium*, *Klebsiella*, and *Campylobacter* all had high abundance on corn without a seed coating agent, but low abundance on corn with a seed coating agent. These bacteria are of great interest in studying both different host plants and different treatments of the same host plant.

**Figure 4:**
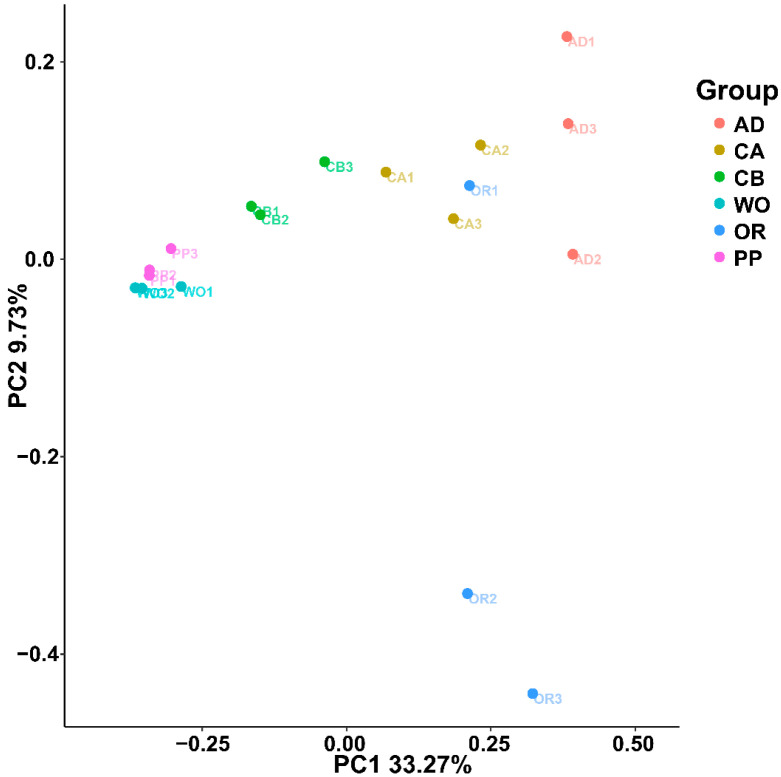
Two-dimensional PCoA analysis visualization using the Bray–Curtis distance calculation method to measure different host samples. The abscissa (PC1) and ordinate (PC2) are the two main coordinates with the largest interpretative degree of differences between samples. The same color represents the same grouping. A point is a sample, and similar samples are gathered together.

Through PCoA analysis based on the Bray–Curtis distance matrix algorithm, combined with the significantly different *p*-values of Adonis analysis, the significant differences in microbial community distribution in samples of different hosts were compared, and the differences in multiple sets of data were reflected by a two-dimensional coordinate diagram (Figure 4). In the scatterplot, the X-coordinate PC1 explains 33.27% of the data changes, while the Y-coordinate PC2 explains 29.7% of the changes. As shown in the figure, the host samples were essentially clustered together, without obvious overlap. This, combined with the results of ANOSIM analysis, shows that the gut microbial community structure of each host was significantly different (r = 0.503, *p* = 0.001). The microbial communities of wild oat and pepper were the most similar, and there were differences between the two kinds of corn, while artificial diets were markedly different from other hosts.

**Figure 5:**
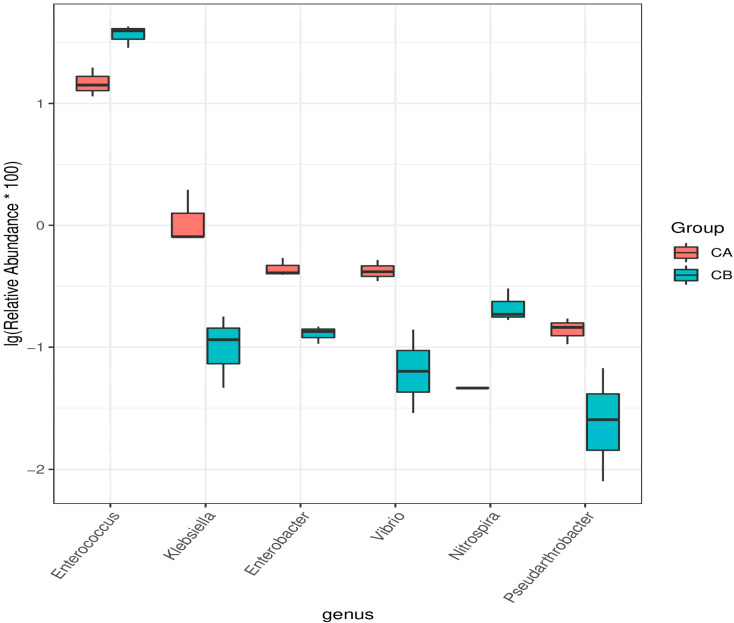
Relative abundance of genera that showed significant differences between corn with a seed coating agent and corn without a seed coating agent. Welch’s t-test was used to evaluate the differences. The genera were significantly different between the two hosts (*p* < 0.05).

In order to further understand the microbial communities of corn with and without a seed coating agent, the microbial genera with significant differences between the two groups were compared (Figure 5). The six genera in the figure showed significant differences between the two groups. The relative abundance of *Enterococcus* was the highest. The relative abundance of *Klebsiella*, *Enterobacter*, *Vibrio*, and *Pseudarthrobacter* on corn without a seed coating agent was greater than that on corn with a seed coating agent. However, the relative abundance of *Enterococcus* and *Nitrospira* on the latter was higher than that on the former.

**Figure 6:**
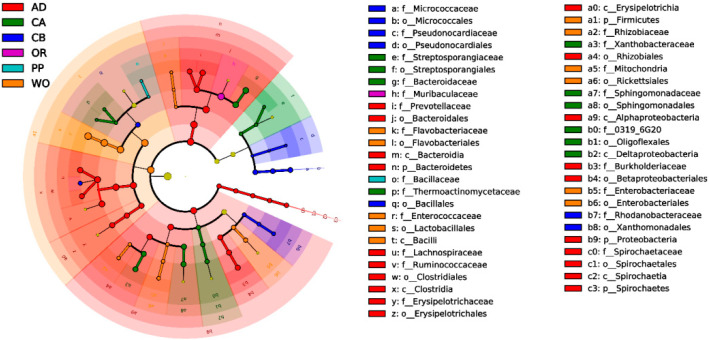
Cladogram of bacterial biomarkers, from the phylum (innermost ring) to genus (outermost ring) levels. The circles at different taxonomic levels represent a taxon at that level, and the relative abundance of the flora is represented by the diameter. Insignificantly different species are yellow, and different colors represent different species. The colors represent the groups, and the different colored nodes represent the bacteria that play an important role in that group.

According to the distribution of LDA (linear discriminant analysis) values, the main microbial community of different taxonomic orders (kingdom to species) among each host group was analyzed by LEfSe (LDA effect size). Biomarkers with inter-group differences were obtained for each host (Figure S3). To further understand the evolutionary ability of the microbial community of each host and classification level distribution, a cladogram of the bacterial community was drawn, and the longest branch was from phylum to genus (Figure 6). The main gut microbial community of FAWs feeding on an artificial diet included Firmicutes, Proteobacteria, Bacteroidetes, and Spirochetes. There were 13 and 9 microbial species feeding on corn without a seed coating agent and corn with a seed coating agent, respectively, which mainly included Firmicutes, Proteobacteria, and Actinobacteria. Bacteroidetes were unique to corn without a seed coating agent. There were 11 microbial species feeding on wild oat, which contained Firmicutes, Proteobacteria, and Bacteroidetes. There were two microbial species feeding on pepper, which were distributed in the family Bacillaceae of Firmicutes. There was only one microbial species feeding on oilseed rape, which was distributed in the family Muribaculaceae of Bacteroidetes. Based on the LEfSe analysis and cladogram, the effects of feeding on different hosts on the gut microbial community of FAWs are further explained. The experimental results of this study are consistent with the conclusions drawn from other lepidopteran herbivores.

### 2.3. Functional Prediction of Microbiota

**Figure 7:**
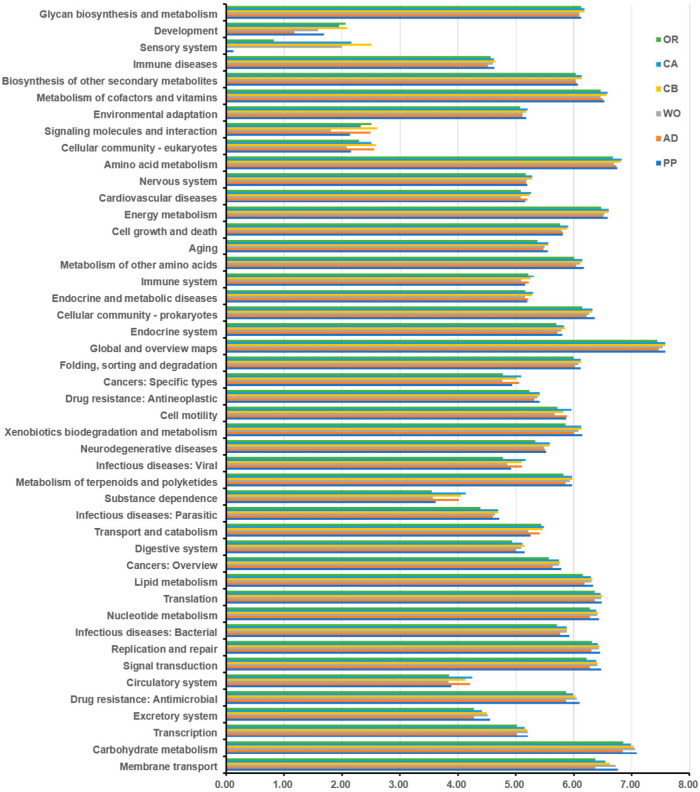
Comparison of KEGG function prediction of feeding on each host.

**Figure 8:**
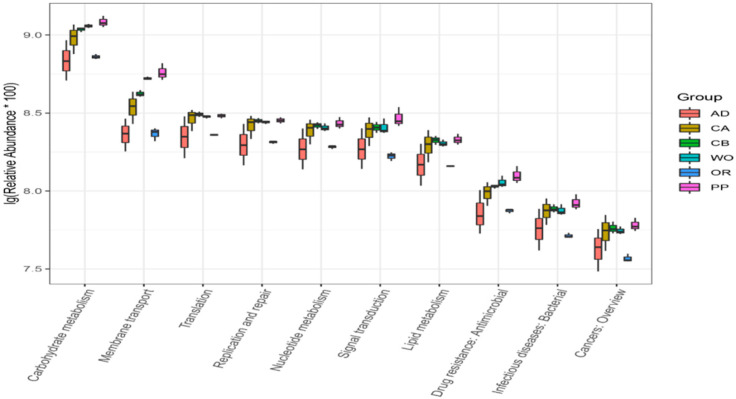
Top 10 functional predictions with significant differences between hosts. There were significant interspecific differences in the 10 functions of each host.

In order to further understand the role of the gut microbial community of the FAW, the sequenced 16S rDNA data were used to predict functional genes via PICRUSt2, and these results were compared with a KEGG database. As shown in Figure 7, 46 functions were predicted, most of which were related to metabolic and cellular processes. The main metabolic functions included sugar synthesis and metabolism, carbohydrate transport and metabolism, amino acid transport and metabolism, inorganic ion transport and metabolism, energy generation and conversion, biodegradation and metabolism, transcription, replication, recombination and repair, drug resistance, nervous system, immunity, growth and development, etc. All of these predicted pathways perform the most important functions in the gut, and play a vital role in the overall growth and development of the FAW. In order to further analyze the relationships between host and function, the top 10 predicted functions with significant differences in feeding on each host were analyzed in the reanalysis (Figure 8). Differences in feeding hosts lead to differences in carbohydrate metabolism, followed by membrane transport, translation, replication and repair, nucleotide metabolism, signal transduction, lipid metabolism, drug resistance, and cancer. We can thus see that feeding on different hosts can lead not only to differences in metabolism within the body, but also to differences in resistance to drugs and cancer. These pathways are the most important functions to maintain the growth, development, and population reproduction of the FAW.

## 3. Discussion

Microbes, as special commensals, exist in the host body and play an important role in host growth and development, ecological changes, invasion changes, and evolution [18,47]. The research on gut microbes is quite extensive, and insects are suitable hosts of microbes. In recent years, research on the gut bacteria of insects has gradually deepened. The interactions between gut bacteria and insect hosts are complex, involving morphology, behavior, physiology, and biochemistry. Insects are metamorphosed developmental organisms with a long growth cycle in the larval stage and stable overall growth and development, and are thus suitable for studying gut bacteria. Although the harsh gut conditions of lepidopteran larvae are not as suitable for microbial life [17,50], it is important to consider that the FAW is a highly polyphagous invasive pest that feeds on a wide range of hosts. In this experiment, the composition of the microbial communities of FAWs fed on different hosts was studied. The microbial abundance on pepper and the microbial diversity on wild oat were the lowest, suggesting that Solanaceae and Gramineae provide different nutrients for the growth and development of FAWs. The study of Wu et al. [4] showed that among solanaceous vegetables, FAWs feeding on pepper had the longest pre-adult period and the lightest pupal weight. On oilseed rape, the high abundance and diversity in the gut microbial community may contribute to the absorption of specific nutrients from unbalanced feeding, and to the adaptation of FAWs to the feeding environment [18,47]. He et al. [2] noted that among oil crops in China, FAWs feeding on oilseed rape had the highest pupa mass—significantly higher than that of other crops—and the lowest average generation period. The abundance and diversity in the gut microbial community on corn without a seed coating agent were higher than those on corn with a seed coating agent, indicating that feeding on corn without a seed coating agent is more suitable for the growth and development of FAWs.

At the phylum level, Firmicutes, Bacteroidetes, Proteobacteria, and Actinomycetes were prevalent in all samples, and were the dominant microbial communities, similar to the results of diversity and dynamics for *Nilaparvata lugens* [51], *Saperda vestita* [52], and *Reticulitermes speratus* [53]. Firmicutes and Proteobacteria were the dominant phyla in many insect samples [17,18,25,47,54]. Firmicutes are abundant in the digestive tracts of *Spodoptera litura*, *Manduca sexta*, *Helicoverpa armigera*, and several other lepidopterans [17]. Absolute dominance in the abundance of Proteobacteria in *G. molesta* [18], *Bombyx mori* [23,54], *N. lugens* [25,51,55], mosquitoes [42,56], and *Triatoma sordida* [57] has been confirmed. The high abundance of Firmicutes on wild oats is due to better absorption of different nutrients. Previous studies have shown that Proteobacteria can degrade pesticides and plant secondary substances. Proteobacteria and Firmicutes, which are highly abundant, play an important role in host environment adaptation, digestion, nutrient utilization, and energy metabolism [18,47]. Enterococcaceae are a special family of microorganisms, with high abundance on wild oat and pepper—significantly higher than on other hosts—and low abundance on artificial diets and oilseed rape. It is interesting to note that the abundance of important microbes varied by hundreds of times in the same batch of experiments. Enterococcaceae are capable of degrading alkaloids and latex, and play a stabilizing role in tolerant plants [47,58]. *Enterococcus* is also found in other lepidopteran insects, such as *C. pomonella* [47], *Lymantria dispar* [59], *S. litura* [33,60,61], *Conogethes punctiferalis* [47], *M. sexta* [62], and *G. molesta* [47]. Artificial diets are relatively rich in nutrients and low in alkaloid latex, requiring few bacteria to participate in the degradation process, while other host plants are just the opposite. Muribaculaceae are a new family of S24-7 (Bacteroidetes) that have not yet been named. Muribaculaceae have not been reported in the gut microbial communities of insects. Studies have shown that Enterobacteriaceae play a role in the metabolism of sugar in larvae, and researchers speculate that they also play a role in digestion, protection, courtship, and reproduction [26,47,63]. *Clostridia* can degrade cellulose and metabolize amino acids to obtain nutrients from the host [18,64]. Burkholderiaceae and *Pseudomonas* can metabolize insecticides [18,65,66,67].

There were significant differences in gut microbial community structures between different hosts. There was some overlap between pepper and wild oat in PCoA, suggesting that pepper and wild oat may provide the special nutrients needed for the growth and development of the FAW. The clustering of the two kinds of corn indicated that although they were the same host plant, different treatments produced obvious differences in gut microbes. These results strongly suggest that feeding on different hosts affects the gut microbial community of insects. The differences in the genus-level structures of microbial communities between the two corn types were compared. The relative abundance of *Enterobacter* in corn without a seed coating agent was greater than that in corn with a seed coating agent; for *Enterococcus*, the situation was the opposite. As mentioned above, *Enterobacter* plays an important role in larval sugar metabolism, digestion, protection, courtship, and reproduction, which indicates that the gut microbe functions are reproduction and population expansion when feeding on corn without a seed coating agent. *Enterococcus* plays an important role in both tolerant and adaptive plants, which indicates that the FAW is not well adapted to this host when feeding on corn with a seed coating agent, and needs further adaptation to this host.

Although there were 46 predicted functions of the gut microbes feeding on different hosts, most of these functions were related to metabolic and cellular processes. They mainly included sugar synthesis and metabolism, carbohydrate transport and metabolism, amino acid transport and metabolism, nervous system and immunity, etc. This conclusion is similar to those of studies of *G. molesta* [18,47,48], which analyzed the significant differences in predicted function between different hosts, and concluded that feeding on different hosts can cause differences in metabolism and resistance to drugs and cancer. Previous studies have shown that the gut microbiota of the FAW can change the metabolic process of insecticides and improve detoxification efficiency under the pressure of pesticide selection [17]. These pathways play an important role in adapting to different hosts and environments, ensuring the normal growth and development of individual insects and maintaining the stability of population reproduction.

In conclusion, high-throughput amplicon sequencing was used to comprehensively study the microbial community present on the host species—namely, Cruciferae, Gramineae, Solanaceae, and artificial diets. The results show that there are significant differences in the community structures of the microorganisms feeding on different hosts, that the interactions among the microorganisms are complex, and that their corresponding functions are not clear. Metagenomic and transcriptomic analyses are required in order to illustrate host–microbiome interactions. In this study, the in-depth understanding obtained of the related microbial communities of FAWs will provide a basis for the development and research of biocontrol technologies based on the complex relationships between insects and symbiotic microorganisms.

## 4. Materials and Methods

### 4.1. Insect Culture

Laboratory trials were carried out at the Northwest A&F University, Yangling, Shaanxi, China. FAW larvae were collected in the field in September 2020 in Xingping (Shaanxi, China) and reared for 5 consecutive generations on corn plants of the same variety in the laboratory. The population was kept at 25 ± 1 °C under a 16:8 h light/dark photoperiod and a relative humidity (RH) of 75 ± 5%. Trials were carried out from the 6th generation, feeding on different hosts.

### 4.2. Host Category

The feeding hosts in this experiment included host plants and artificial diet (AD). Host plants included corn (*Zea mays* L. var. Zhengdan 958, without a seed coating agent (CA) and with a seed coating agent (CB)), wild oat (*Avena fatua*, WO), oilseed rape (*Brassica napus* L. var. Shaanyou 28, OR) and pepper (*Capsicum annuum* L. var. Dejiao 6, PP) (The occurrence of the above six hosts is replaced by abbreviations). The artificial diet contained 600 g of cornmeal, 200 g of dry yeast, 200 g of soybean meal, 5 g of citric acid, 5 g of casein, 8 g of methyl-p-hydroxy benzoate, 5 g of sorbic acid, 3 g of vitamin B complex, 30 g of ascorbic acid, 1 g of roxithromycin, 50 g of agar, 200 μL of propionic acid, and 3200 mL of distilled water [2]. The artificial diet was prepared in the laboratory to ensure its freshness and usability. Plant seeds were purchased from a local agricultural materials company (Yangling, Shaanxi, China). Seeds of respective plants were cultivated singly in potting mix (a mixture of perlite, peat moss, vermiculite, and cow manure–alfalfa mix organic fertilizer in a 1:10:10:10 ratio by volume) in disposable culture bowls in glasshouses. Plants were cultivated to the 3–4-true-leaf stage, at which time they were used in the experiments [2].

### 4.3. Sample Collection and DNA Extraction

FAW larvae were fed on different hosts until they reached the 5th instar for experiment sample collection [17]. When larvae achieved the specific instar, they were rinsed with 0.5% sodium hypochlorite, 70% ethanol, and sterile water, and surface-sterilized successively 3 times, for 30–60 s each time. The whole sampling process was carried out under a sterile environment. Guts were dissected in a Petri dish with sterilized surgical forceps on ice under a stereomicroscope, and the samples were temporarily stored in precooled phosphate-buffered saline (PBS; pH 7.4; 140 mmol/L NaCl, 2.7 mmol/L KCl, 10 mmol/L Na_2_HPO_4_, 1.8 mmol/L KH_2_PO_4_) [68,69]. Fifteen guts were taken as one sample, and each sample had three replicates.

Genomic DNA (gDNA) from samples was extracted using the MagPure Soil DNA LQ Kit (D6356-02, Omega, Magen Inc., New York, NY, USA), according to the manufacturer’s detailed protocol [17,18,47]. DNA quantity and the quality of the samples obtained were measured using a NanoDrop 2000 spectrophotometer (Thermo Fisher Scientific, Waltham, MA, USA). The integrity was evaluated via agarose gel electrophoresis within ethidium bromide in TAE buffer [18,47]. The gDNA was then stored at −80 °C for subsequent experiments.

### 4.4. PCR Amplification and High-Throughput Sequencing

The effects of different host feeding on microbial community composition and diversity, as well as FAWs, were analyzed through bacterial diversity analysis of the V3-V4 region of the 16s rRNA gene, amplified using the Illumina MiSeq platform [17,18]. Primers included 343F (5′-TACGGRAGGCAGCAG-3′) and 798R (5′-AGGGTATCTAATCCT-3′) [70,71]. PCR reactions were carried out with Tks Gflex DNA Polymerase (TaKaRa, San Jose, CA, USA), performed in a total volume of 30 µL, containing 15 µL of 2 × Gflex PCR Buffer, 1 µL of each primer (10 µmol/L), 0.6 µL of Tks Gflex DNA Polymerase (1.25 U/μL), 1 µL of template DNA (about 50 ng), and 11.4 µL of ddH_2_O in a thermocycler programmed at 94°C for 5 min (1 cycle); 94 °C for 30 s; 56 °C for 30 s; 72 °C for 20 s (35 cycles); 72 °C for 5 min (1 cycle), with a final hold at 4 °C [17,51]. Prior to high-throughput sequencing, purified PCR amplification efficiency was confirmed with 2% agarose gel electrophoresis and purified using Agencourt AMPure XP beads (Beckman, Brea, CA, USA). Ultrapure water was used as a blank control to exclude the possibility of non-PCR results. Finally, the 16S rRNA high-quality amplicon fragments quantified from each sample with 10 ng were pooled and subsequently subjected to the Illumina MiSeq platform for sequencing with the PE300 model of Shanghai Oe Biotech Co., Ltd. (Shanghai, China) [18,47,51].

### 4.5. Statistical and Bioinformatics Analysis

According to the criteria of overlap greater than 10 bp and a mismatch rate less than 0.02, combined with the unique barcode of each sample, FLASH (version 1.2.11, Baltimore, MD, USA) was used to truncate the barcode and primer sequences and reassign the paired-end reads [51,68,72,73,74]. Low-quality sequences that were shorter than 250 bp or contained ambiguous bases were discarded. Chimeric sequences were detected and removed using the UCHIME (version 2.4.2, http://drive5.com/usearch/manual/uchime_algo.html) algorithm, and valid tags were finally obtained [75]. The PyNAST method was used to effectively align the sequences in the SILVA123 (http://www.arb-silva.de) or Greengenes databases [17,76,77]. Sequence analyses were performed via the UCLUST method (http://drive5.com/usearch/manual/uclust_algo.html) [76]. The remaining high-quality sequences were classified into OTUs, considering a 97% similarity truncation value. The OTU sequences with the highest relative abundance were selected for screening, and the overall OTU table was constructed and further annotated based on the RDP Classifier (Version 2.2, https://sourceforge.net/projects/rdp-classifier) [68,78]. We combined sequences with less than 1% abundance and replaced the data with “other”. The phylogenetic relationships of each OTU and the characteristics of dominant species in each group (sample) were studied using the MUSCLE (http://www.drive5.com/muscle) routine for multi-sequence alignment [79]. OTU abundance information was normalized according to the sequence number corresponding to the sample with the smallest sequence. Subsequent analyses of alpha diversity and beta diversity were performed on the basis of this output standardized data.

We performed analysis of alpha diversity and beta diversity indices using QIIME V1.7.0 (http://qiime.org) [51]. Alpha diversity analysis mainly referred to the Chao1 index and Shannon index to evaluate species abundance and diversity. Beta diversity usually compared microorganism similarity between samples via principal coordinate analysis (PCoA) [68]. We used the R vegan package to obtain a heatmap showing the differences in genus abundance profiles between samples [47]. The taxonomic changes among different groups were analyzed via STAMP (https://beikolab.cs.dal.ca/software/STAMP). The functional abundance of microorganisms in the samples was predicted using the KEGG databases with PICRUSt (https://sourceforge.net/projects/picrust) [47,80]. Based on the assumptions of normality and homogeneity of variance, ANOVA followed by LSD and Tukey’s HSD tests were used for data analysis. All trial data were analyzed using SPSS Statistics 26.0 (https://www.ibm.com/products/spss-statistics). Differences were considered significant when * *p* < 0.05, and extremely significant when ** *p* < 0.01.

## Figures and Tables

**Table 1:** Relative abundance of dominant bacteria at the phylum and family taxonomic levels of different hosts.

Taxonomy	Group	AD	CA	CB	WO	OR	PP
Phylum	Firmicutes	21.99 ± 1.14% ^d^	34.31 ± 1.91% ^cd^	50.61 ± 3.46% ^bc^	74.05 ± 7.76% ^a^	29.26 ± 4.51% ^d^	65.84 ± 3.65% ^ab^
	Bacteroidetes	45.82 ± 4.22% ^ab^	36.52 ± 2.92% ^ab^	27.39 ± 3.19 ^bc^	11.24 ± 1.84% ^c^	54.28 ± 9.88% ^a^	11.17 ± 1.44% ^c^
	Proteobacteria	22.16 ± 1.80% ^a^	18.70 ± 1.18 ^a^	13.34 ± 1.19% ^a^	11.0.9 ± 5.43% ^a^	10.30 ± 2.97% ^a^	19.32 ± 4.99% ^a^
	Actinobacteria	3.59 ± 0.165 ^ab^	4.32 ± 1.18% ^a^	4.55 ± 0.88% ^a^	1.58 ± 0.22% ^b^	2.57 ± 0.85% ^ab^	1.38 ± 0.13% ^b^
	Total	93.47 ± 0.88% ^a^	93.85 ± 1.73% ^a^	95.88 ± 0.50% ^a^	97.96 ± 0.42% ^a^	96.41 ± 2.10% ^a^	97.70 ± 0.37% ^a^
Family	Enterococcaceae	0.27 ± 0.01% ^d^	15.44 ± 2.17% ^c^	37.24 ± 4.19% ^b^	67.76 ± 9.12% ^a^	8.53 ± 3.44% ^cd^	58.71 ± 3.11% ^a^
	Muribaculaceae	27.09 ± 3.68% ^ab^	19.27 ± 2.53% ^ab^	14.10 ± 1.53% ^b^	6.09 ± 0.97% ^b^	40.10 ± 10.28 ^a^	6.49 ± 0.85% ^b^
	Enterobacteriaceae	6.10 ± 0.45% ^a^	5.69 ± 0.52% ^a^	3.35 ± 0.60% ^a^	6.64 ± 4.91% ^a^	3.82 ± 0.76 ^a^	14.76 ± 5.33% ^a^
	Lachnospiraceae	7.79 ± 0.62 ^ab^	7.97 ± 1.155 ^a^	3.84 ± 0.21% ^bc^	2.24 ± 0.57% ^c^	7.88 ± 1.455 ^ab^	2.71 ± 0.52% ^c^

Mean ± SE. Different letters (a, b, c, d) indicate that the same bacterium has significant differences between different host plants (*p* < 0.05; ANOVA with Tukey’s HSD test).

## Data Availability

The data presented in this study are available in the Supplementary Materials.

## Supplementary Materials

The following are available online at https://www.mdpi.com/article/10.3390/ijms222011266/s1.
