# Supplementary material for: Comparison of Gut Bacterial Communities of Fall Armyworm (Spodoptera frugiperda) Reared on Different Host Plants
Source: Int J Mol Sci. 2021 Oct 19;22(20):11266. doi: 10.3390/ijms222011266 (PMC8540368; doi:10.3390/ijms222011266)
Supplement: Supplementary file 1 [file ijms-22-11266-s001.zip › Supplementary Figure S2.pdf]

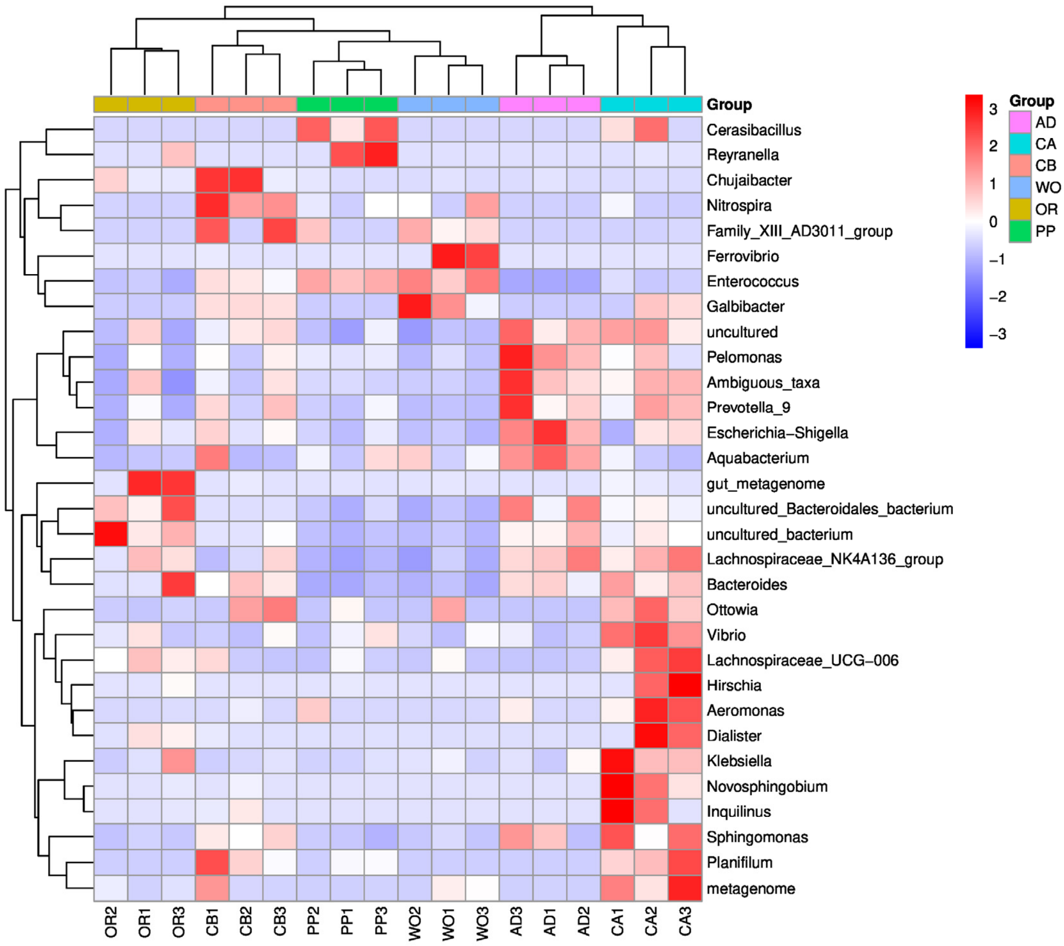

Figure S2. Heatmap of dominant bacteria in different samples at the level of genus classification. Transverse is sample information. Longitudinal is species annotation information. The cluster tree on the left of the figure is a species cluster tree. The cluster branch group on top represents samples from different hosts. Red indicates relatively high species abundance, and blue indicates relatively low species abundance.
