# Supplementary material for: Comparison of Gut Bacterial Communities of Fall Armyworm (Spodoptera frugiperda) Reared on Different Host Plants
Source: Int J Mol Sci. 2021 Oct 19;22(20):11266. doi: 10.3390/ijms222011266 (PMC8540368; doi:10.3390/ijms222011266)
Supplement: Supplementary file 1 [file ijms-22-11266-s001.zip › Supplementary Table S1.pdf]

Table S1. Summary of sequence and OTUs data for the Illumina MiSeq operation of the experimental samples

Sample ID	Clean tags	Valid tags	Valid percent	Valid min length	Valid mean length	Valid max length	OTU counts	Total OTUs
AD1	46923	42209	89.95%	229	408.99	443	657	6452
AD2	68920	61503	89.24%	250	415.9	457	851	6452
AD3	33892	30933	91.27%	252	411.09	443	670	6452
CA1	69308	60618	87.46%	258	415.63	458	1112	6452
CA2	53604	48134	89.80%	237	414.95	443	769	6452
CA3	66751	60503	90.64%	229	412.54	449	951	6452
CB1	67171	62421	92.93%	256	418.75	443	776	6452
CB2	68492	59072	86.25%	256	417.17	443	1281	6452
CB3	69848	63926	91.52%	255	417.89	443	688	6452
WO1	68619	64456	93.93%	233	421.51	442	822	6452
WO2	69829	65979	94.49%	255	423.16	443	767	6452
WO3	69080	64587	93.50%	256	422.23	443	702	6452
OR1	49247	44661	90.69%	236	415.67	442	836	6452
OR2	69107	63280	91.57%	236	417.2	443	1289	6452
OR3	71996	60821	84.48%	223	374.72	441	2235	6452
PP1	70113	66957	95.50%	252	423.08	448	662	6452
PP2	70957	67211	94.72%	256	421.84	455	669	6452
PP3	68222	63903	93.67%	258	421.24	451	642	6452
