# Supplementary material for: Comparison of Gut Bacterial Communities of Fall Armyworm (Spodoptera frugiperda) Reared on Different Host Plants
Source: Int J Mol Sci. 2021 Oct 19;22(20):11266. doi: 10.3390/ijms222011266 (PMC8540368; doi:10.3390/ijms222011266)
Supplement: Supplementary file 1 [file ijms-22-11266-s001.zip › Supplementary Table S2.pdf]

Table S2. Alpha diversity index and number of species observed

Samples	Chao1	Shannon	Simpson	PD_whole_tree	Good's_coverage	Observed_species
AD1	1028.51	7.6702	0.9911	30.3134	0.9928	576.60
AD2	1123.51	8.0691	0.9926	34.7304	0.9933	684.00
AD3	1029.97	7.7522	0.9918	32.0139	0.9923	664.20
CA1	1294.20	7.6731	0.9581	47.0583	0.9938	962.30
CA2	1162.03	7.3596	0.9810	31.5851	0.9915	646.20
CA3	1211.50	7.8387	0.9759	38.1084	0.9935	792.30
CB1	970.64	5.5406	0.8143	30.6252	0.9936	601.20
CB2	1458.89	6.2261	0.8415	49.5659	0.9917	1096.80
CB3	884.12	6.5019	0.9151	29.0913	0.9944	530.40
WO1	996.46	4.5854	0.7418	33.4088	0.9936	637.20
WO2	935.23	2.7332	0.4260	29.6986	0.9934	567.10
WO3	848.52	2.5908	0.3977	29.2599	0.9944	559.20
OR1	1140.24	7.7299	0.9783	36.4895	0.9924	740.70
OR2	1397.63	7.0110	0.9521	45.9229	0.9923	1099.30
OR3	2359.13	7.6192	0.9809	64.4062	0.9800	1841.00
PP1	902.05	3.5125	0.6839	28.0981	0.9945	495.20
PP2	847.84	3.5383	0.6002	28.3418	0.9944	507.00
PP3	824.65	3.8014	0.6272	25.2728	0.9945	485.80
