# Supplementary figures and images for: Comparison of Gut Bacterial Communities of Fall Armyworm (Spodoptera frugiperda) Reared on Different Host Plants
Source: Int J Mol Sci. 2021 Oct 19;22(20):11266. doi: 10.3390/ijms222011266 (PMC8540368; doi:10.3390/ijms222011266)

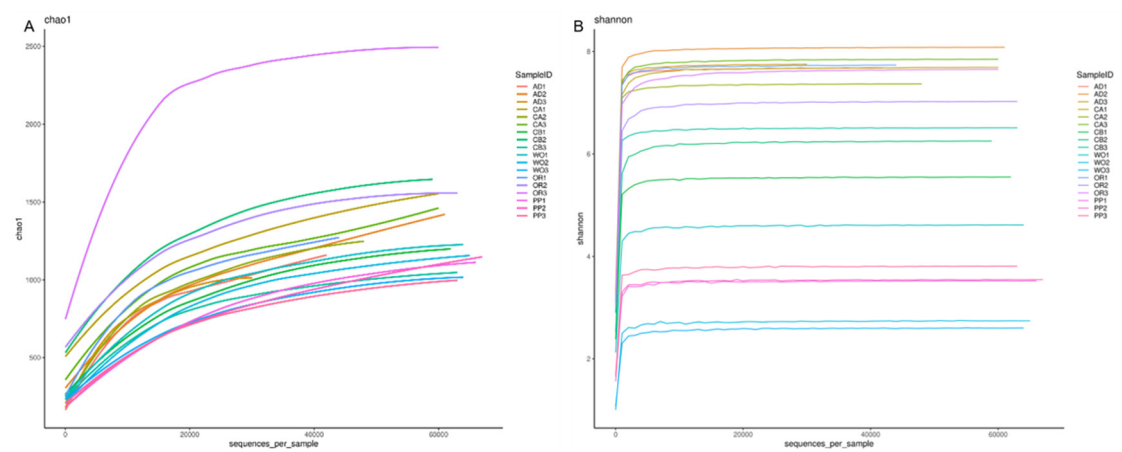

**Figure S1. Alpha diversity index rarefaction curves. (A) Chao1 index (B) Shannon index**

Supplement: Supplementary file 1 [file ijms-22-11266-s001.zip › Supplementary Figure S1.pdf]

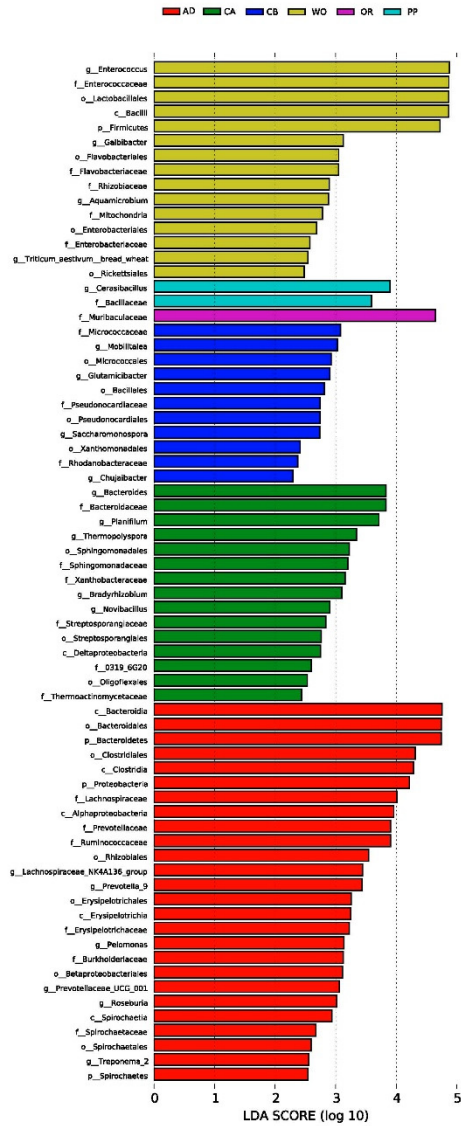

**Figure S3. Bacterial taxa with linear discriminant analysis (LDA) score of each host**

Supplement: Supplementary file 1 [file ijms-22-11266-s001.zip › Supplementary Figure S3.pdf]
